# Astaxanthin and Related Carotenoids as a Dual Modulator of Nrf2 and NF-κB in Bisretinoid-Induced Retinal Damage: Mechanistic Rationale and Evidence Gaps

**DOI:** 10.3390/antiox15101273

**Published:** 2026-10-02

**Authors:** Babita Kumari Baniya, Hye Jin Kim

**Affiliations:** College of Pharmacy, Keimyung University, 1095 Dalgubeol-daero, Dalseo-gu, Daegu 42601, Republic of Korea; babita8@kmu.kr

**Keywords:** astaxanthin, Nrf2, NF-κB, bisretinoid, A2E, retinal pigment epithelium, age-related macular degeneration

## Abstract

The retinal pigment epithelium (RPE) plays a key role in age-related macular degeneration (AMD). With aging, bisretinoids such as A2E accumulate in RPE lysosomes, and this accumulation is markedly accelerated by ABCA4 loss of function, which impairs the clearance of retinaldehyde from photoreceptor disc membranes. A2E acts as a blue-light photosensitizer, generating reactive oxygen species that cause oxidative stress, lysosomal impairment, and mitochondrial dysfunction. This oxidative burden disrupts the balance between Nrf2-mediated antioxidant defense and NF-κB-driven inflammation. Astaxanthin, a marine-derived xanthophyll carotenoid, may regulate this balance by activating Nrf2/ARE-mediated cytoprotective responses and suppressing NF-κB-dependent inflammation. This review summarizes mechanistic, preclinical, and clinical evidence for astaxanthin’s protective effects on RPE cells, with emphasis on bisretinoid-induced retinal injury. Astaxanthin reduces oxidative stress, inhibits NF-κB activity, and enhances Nrf2-dependent antioxidant defenses in hydrogen peroxide-, light-, and ischemia-induced retinal damage; however, because these models do not directly reproduce A2E-mediated phototoxicity, A2E-specific protection remains an untested hypothesis. Standardized A2E-specific models are needed to determine whether these protective effects extend to A2E and its photoproducts. Clinical evidence is also limited and largely based on multi-carotenoid formulations, which prevents the isolation of the effects of astaxanthin.

## 1. Introduction

The retina is vital for vision. However, its high metabolic activity, continuous exposure to light, and lipid-rich composition make the retina especially vulnerable to oxidative stress. This vulnerability can cause retinal malfunction and gradual vision loss [1]. The retinal pigment epithelium (RPE), a highly specialized and distinct epithelial cell, interacts with photoreceptors on its apical side and with Bruch’s membrane and the choriocapillaris on its basal side. The RPE plays a crucial role in vision by supporting photoreceptors, but aging and environmental stress can impair its function, leading to retinal degeneration and vision loss [2]. One of the main causes of permanent vision loss globally, age-related macular degeneration (AMD), is characterized by degeneration of RPE cells [2,3]. Autofluorescent lipofuscin gradually accumulates in RPE cells, a sign of aging and several hereditary retinal diseases [4]. Bisretinoids, including A2E, are important lipofuscin fluorophores that are implicated in the pathogenesis of AMD [5,6]. A2E functions as a photosensitizer, producing reactive oxygen species (ROS) when exposed to blue light. This causes oxidative stress, lysosomal dysfunction, and mitochondrial damage, which in turn accelerates the development of AMD and RPE degeneration [7]. The detailed mechanism underlying A2E biosynthesis, lysosomal accumulation, and retinal toxicity is discussed in Section 3.

This framing is supported by two recent reviews published in Antioxidants: one places complement activation and oxidative stress-driven mitochondrial dysfunction at the center of AMD onset and progression [8], while another emphasizes lysosomal dysfunction along with impaired Nrf2/TFEB signaling as an emerging, mechanistically proximal target in the aging RPE, directly related to the lysosomal handling of A2E covered in Section 3 [9].

Excess ROS and compromised antioxidant defenses caused by oxidative stress and inflammation cause retinal degeneration, which results in RPE impairment, mitochondrial damage, immunological dysregulation, and vision loss [10]. Retinal degeneration is cooperatively regulated by Nrf2-mediated antioxidant defense and nuclear factor kappa B (NF-κB)-driven inflammation. An imbalance between these processes results in RPE damage, persistent inflammation, and vision loss [10,11]. Apart from directly harming cells, oxidative stress triggers inflammatory signaling pathways, specifically the NF-κB pathway, which promotes the production of pro-inflammatory cytokines such as TNF-α, IL-1β, and IL-6. As a result, reducing inflammation and oxidative stress has become a viable method of stopping retinal degeneration [12,13]. Astaxanthin is a red, fat-soluble xanthophyll carotenoid that is present in a variety of microorganisms and marine creatures. It has stronger biological activity than other carotenoids but does not have provitamin A activity in humans [14]. It reduces oxidative stress in RPE cells, efficiently scavenging ROS [15]. More broadly, astaxanthin suppresses NF-κB-mediated inflammatory response and boosts cellular antioxidant defenses by activating Nrf2 signaling. Astaxanthin uses this dual regulatory mechanism to prevent oxidative damage to RPE cells, reduce inflammation, and potentially halt the advancement of AMD [16,17].

## 2. Literature Search Strategy

### 2.1. Search Terms and Databases

A structured literature search was conducted in PubMed, Scopus, Google Scholar, and ScienceDirect from database inception to 22 September 2026, to identify potentially relevant studies. The search strategy was formulated using the following keywords as search terms: (“age-related macular degeneration” OR “dry age-related macular degeneration” OR “non-neovascular age-related macular degeneration” OR “non-exudative age-related macular degeneration”) AND (“oxidative stress” OR “reactive oxygen species” OR “A2E” OR “A2E bisretinoid” OR “bisretinoids” OR “lipofuscin” OR “retinal pigment epithelium” OR “RPE” OR “mitochondrial dysfunction” OR “ER stress” OR “Nrf2” OR “NF-κB” OR “antioxidant” OR “dietary supplements” OR “marine carotenoid” OR “astaxanthin”). To identify non-retinal mechanistic studies examining the antioxidant, anti-inflammatory, and NF-κB-related effects of astaxanthin in pertinent cellular and animal models, including acute lung injury, gastric epithelial cells, macrophages, and other experimental models, additional targeted searches were conducted in addition to the primary retinal/AMD-focused search. Retrieved articles were screened for those assessing antioxidant, anti-inflammatory, and mechanistic effects relevant to dry AMD and related oxidative stress conditions. Relevant studies identified during the peer-review process were also considered and incorporated into the review where appropriate.

### 2.2. Eligibility Criteria

The following eligibility criteria were set for the inclusion of the articles in the study: (i) preclinical and clinical studies; (ii) studies investigating antioxidant, anti-inflammatory, cytoprotective, or related molecular mechanisms relevant to dry AMD, retinal oxidative stress, or other non-retinal models providing mechanistic evidence relevant to the review; (iii) articles published in English. Conference abstracts, proceedings, and in silico studies were excluded. An explicit extrapolation boundary was applied because mechanistic studies conducted outside the retina are included. Non-retinal and cell-free findings are only used to demonstrate that a molecular mechanism is chemically and biologically plausible, never as proof that the mechanism functions in the RPE or provides protection against bisretinoid photooxidation. In the evidence table in Section 7, these investigations are classified as non-retinal, and the restriction is reiterated in the section that discusses them.

### 2.3. Data Extraction and Analysis

The titles and abstracts were initially screened for the eligibility criteria, followed by a detailed full-text evaluation considering the listed inclusion criteria. The chosen papers were used to extract pertinent data, such as experimental models, interventions, biological pathways, and reported outcomes. Any discrepancies in the eligibility of the study or data extraction were addressed through discussions.

Methodological quality of the in vivo studies included in the evidence grading in Section 7 was evaluated using the SYRCLE risk-of-bias tool [18] and the CAMARADES study-quality checklist [19]. All 10 SYRCLE risk-of-bias domains and all 10 CAMARADES quality-checklist items were scored for each in vivo study, with domain-level judgments provided in Supplementary Table S1. In vitro and cell-free studies were not appraised with these tools and are graded only by A2E relevance. Key methodological features, including randomization, allocation concealment, blinded outcome assessment, and a priori sample-size calculation, were rarely reported, resulting in unclear or potentially high risk of bias across one or more domains. These limitations are reflected in the evidence-strength statements and in the footnote to the evidence table in Section 7 and limit quantitative comparisons of protective effects between models. The A2E relevance grade indicates the biological closeness of each model to bisretinoid pathology and is separate from methodological quality. Therefore, the two assessments should be considered independently.

## 3. Bisretinoids and Retinal Pigment Epithelial Damage

Vitamin A-derived fluorophores called bisretinoids are produced naturally as byproducts of the visual cycle and progressively build up in RPE cells with aging. ATP-binding cassette subfamily A member 4 (ABCA4) loss of function accelerates this process by delaying the clearance of retinaldehyde from photoreceptor disc membranes, contributing to early bisretinoid accumulation in recessive Stargardt disease [20]. Bisretinoid formation is initiated in photoreceptor outer segments when retinaldehyde and phosphatidylethanolamine (PE) spontaneously and non-enzymatically condense. A2E was the first bisretinoid identified from human eyes and is among the most extensively studied bisretinoids; dozens of bisretinoid species are now known [21,22].

**Figure 1:**
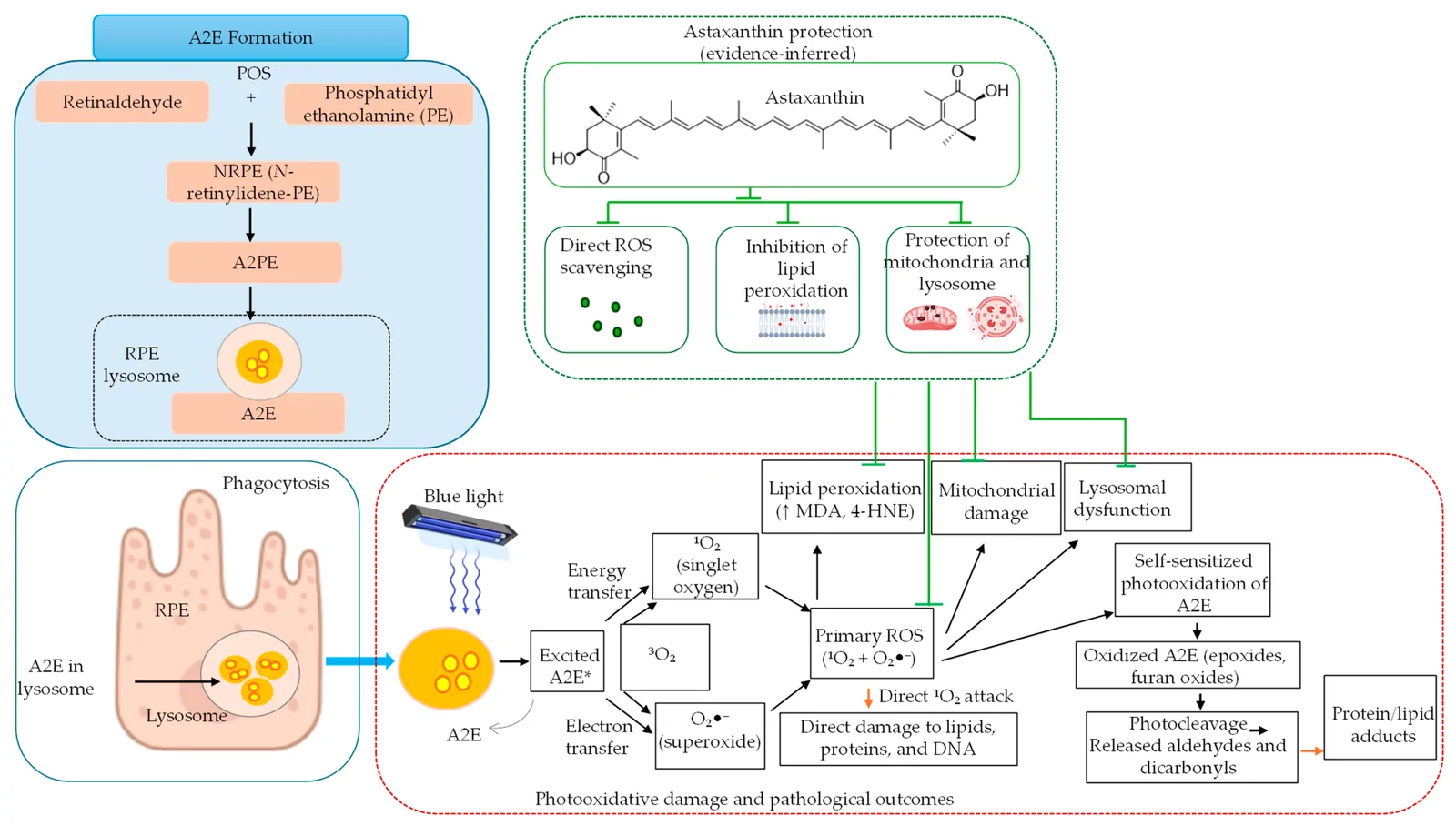
A2E is formed from retinaldehyde and phosphatidylethanolamine (PE) via the intermediates NRPE and A2PE and accumulates in RPE lysosomes after the phagocytosis of photoreceptor outer segments (POS). Blue light excites A2E to A2E*. Excited A2E* generates primary ROS through two photochemical routes: energy transfer to triplet ground-state molecular oxygen (^3^O_2_) produces singlet oxygen (^1^O_2_), whereas electron transfer produces superoxide (O_2_•^−^). From the primary ROS node, two major routes diverge: (i) ^1^O_2_ addition to the A2E polyene chain initiates self-sensitized photooxidation of A2E, producing oxidized A2E species (including epoxides and furan oxides); subsequent photocleavage releases aldehydes and dicarbonyls that form protein/lipid adducts, which are major carriers of toxicity; and (ii) direct ^1^O_2_ attack damages cellular lipids, proteins, and DNA. Primary ROS also contribute independently to lipid peroxidation (↑MDA, 4-HNE), mitochondrial damage, and lysosomal dysfunction, which represent parallel downstream consequences of photooxidative stress. Astaxanthin scavenges primary ROS, particularly singlet oxygen, inhibits lipid peroxidation, and protects mitochondria and lysosomes, thereby interrupting multiple steps in the photooxidative damage cascade. Abbreviations: A2E*, excited-state A2E; A2PE, A2-phosphatidylethanolamine; NRPE, *N*-retinylidene-phosphatidylethanolamine; PE, phosphatidylethanolamine; POS, photoreceptor outer segments; RPE, retinal pigment epithelium; ROS, reactive oxygen species; ^1^O_2_, singlet oxygen; ^3^O_2_, triplet (ground-state) molecular oxygen; O_2_•^−^, superoxide anion radical; MDA, malondialdehyde; 4-HNE, 4-hydroxynonenal. Legend: Blue solid box outline, A2E formation and lysosomal accumulation pathway; blue solid arrow, established biosynthetic and trafficking route; black solid arrow, direct experimental observation of photoexcitation and primary ROS generation; thin black curved arrow, return of excited A2E* to the ground state; green dashed box, evidence-inferred astaxanthin protection based on mechanistic studies; thick green blunt-ended arrow, astaxanthin-mediated inhibition/protection, including singlet-oxygen scavenging; orange solid arrows, direct toxicity routes from carbonyl products to protein/lipid adduct formation and from direct ^1^O_2_ attack to cellular macromolecular damage; red dashed box, photooxidative damage and pathological outcomes.

*N*-retinylidene-phosphatidylethanolamine (NRPE) is first formed when retinaldehyde combines with PE. A2-phosphatidylethanolamine (A2PE) is then produced by condensation with a second retinaldehyde molecule, which may be either all-*trans*-retinal or 11-*cis*-retinal [6]. A2PE is not hydrolyzed to A2E at this stage; rather, after the RPE phagocytoses shed photoreceptor outer segments, A2PE is transported into RPE lysosomes, where it is hydrolyzed to yield A2E [6,23,24,25]. More broadly, bisretinoids arise from non-enzymatic reactions involving vitamin A aldehydes in photoreceptor cells [6]. The biosynthetic sequence from retinaldehyde and phosphatidylethanolamine (PE) through NRPE and A2PE to A2E, followed by its accumulation in RPE lysosomes, is illustrated in Figure 1.

Further evidence from *Nrl−/−* mice demonstrates that cone visual pigments and photoreceptors can contribute to the production of A2E and lipofuscin [26]. A2E accumulates gradually within RPE lysosomes when shed photoreceptor outer segments are phagocytosed because of its positively charged pyridinium ring, which makes it extremely resistant to enzymatic degradation [4,5,23,24,25]. RPE cells are vulnerable to blue-light-induced photooxidation because A2E and other bisretinoids accumulate within RPE lysosomes. Upon blue-light absorption, these bisretinoids act as photosensitizers and transfer energy or an electron to triplet ground-state molecular oxygen, generating singlet oxygen and superoxide. Singlet oxygen can add to their carbon–carbon double bonds and promote photooxidation and photodegradation. Both energy transfer and electron transfer require molecular oxygen. Energy transfer returns A2E to the ground state, so one A2E molecule can generate singlet oxygen repeatedly. Because singlet oxygen also adds to the polyene chain of A2E, part of the A2E pool is consumed through self-sensitized photooxidation [21,27]. Because singlet oxygen is highly reactive and short-lived and can be quenched by the polyene chains of bisretinoids themselves, downstream damage is more likely to be mediated by the resulting photooxidation and photodegradation products than by singlet oxygen directly [21,27]. Oxidized bisretinoids can subsequently fragment into aldehyde- and dicarbonyl-containing products that may modify cellular proteins and lipids, thereby contributing to RPE damage [21,28,29,30]. In addition to light-driven photooxidation, iron can promote bisretinoid oxidation through Fenton chemistry, generating highly reactive hydroxyl radicals that can oxidize A2E and other bisretinoids [20,21]. Importantly, photodegradation of retinal bisretinoids has also been demonstrated in vivo in mouse models, supporting the relevance of this process to retinal disease [31]. Damaged cellular components gradually accumulate because of the increased oxidative burden, which also impairs lysosomal and autophagic activity and disturbs mitochondrial homeostasis, aggravating RPE dysfunction [7,32]. By reducing Nrf2 activity and maintaining NF-κB activation, chronic oxidative stress upsets the equilibrium between antioxidant and inflammatory signaling. This dysregulation can exacerbate RPE dysfunction, promote inflammatory responses, and contribute to processes implicated in age-related macular degeneration [11,21,33].

Despite the widespread use of A2E-loaded ARPE-19 cells as an in vitro model, A2E biology has also been studied in other RPE systems, demonstrating the conservation of numerous basic responses across models. While outer-segment binding, internalization, and protein degradation were mostly maintained in human D407 RPE cells and rat RPE-J cells, physiological levels of A2E localized to lysosomes and preferentially hindered the destruction of photoreceptor outer-segment phospholipids [34]. In RPE models such as ARPE-19, D407, and primary bovine RPE cells, A2E loading also disrupted cholesterol homeostasis by raising cellular free and esterified cholesterol and hindering cholesterol efflux from late endosomal/lysosomal compartments [35]. When combined, these studies show that while the degree of RPE differentiation, polarization, and pigmentation affects the response’s magnitude, the fundamental effects of A2E accumulation, namely lysosomal and phagolysosomal dysfunction and altered lipid handling, are conserved across immortalized human, rat, and primary RPE models. When comparing A2E studies quantitatively, the model employed should be considered because ARPE-19 cells are weakly pigmented and only partially polarized. Confirmation in polarized primary or induced pluripotent stem cell-derived RPE is still preferred.

The processes linked to A2E photooxidative degradation have been further extended by more recent research. In ARPE-19 cells, blue-light exposure (445 ± 18 nm, 4.43 mW/cm^2^) decreased cell viability, enhanced ROS generation, and triggered caspase-3-associated death, with these effects examined in the presence or absence of A2E. Blue-light exposure was also associated with mitochondrial fragmentation and altered expression of regulators of mitochondrial dynamics, including OPA1, DRP1, and OMA1, in both A2E-loaded and unloaded cells, indicating that blue light itself can disrupt mitochondrial dynamics in RPE cells [36]. Furthermore, it has been demonstrated that exposing A2E-loaded ARPE-19 cells to blue light induces ferroptosis, which is characterized by elevated intracellular Fe2+, inhibition of the SLC7A11–GSH–GPX4 axis, and increased lipid peroxidation. Ferroptotic damage was lessened by GSH supplementation, deferiprone reduction of iron buildup, or ferrostatin-1 inhibition of lipid peroxidation. In line with these cellular observations, ferrostatin-1 treatment enhanced retinal function and decreased RPE/photoreceptor degeneration, and ferroptosis-related alterations were found in the RPE of blue-light-exposed *Abca4−/−Rdh8−/−* mice, a model marked by accelerated bisretinoid accumulation [37]. These results give molecular endpoints that may be pertinent when assessing possible carotenoid-mediated protection and highlight mitochondrial dysfunction and ferroptosis as additional downstream effects of A2E-associated photooxidative stress. However, it is yet unclear if astaxanthin may particularly alter these pathways in RPE cells that are loaded with A2E and exposed to blue light.

## 4. Role of Nrf2 and NF-κB Signaling in Retinal Cells

The cellular response to oxidative stress and inflammatory processes in retinal cells is primarily orchestrated by two regulators, Nrf2 and NF-κB. While NF-κB primarily controls inflammatory reactions, Nrf2 mainly facilitates cellular antioxidant defenses [38]. Age-related decline in Nrf2 signaling, together with increased ROS production under photooxidative stress and A2E accumulation, may collectively impair antioxidant defenses and promote NF-κB-mediated inflammation, thereby contributing to AMD pathogenesis [28,39]. The course of AMD is driven by the antagonistic crosstalk between Nrf2 and NF-κB, which is marked by mutual suppression and a vital target for therapeutic interventions [11,39].

### 4.1. Nrf2 Signaling as a Retinal Antioxidant Defense System

Nrf2 is a redox-sensitive transcription factor that protects cells from oxidative stress. Under basal conditions, Nrf2 is sequestered within the cytoplasm by an adaptor protein, Kelch-like erythroid cell-derived protein with CNC homology (ECH)-associated protein 1 (Keap1), which directs the degradation of Nrf2 via ubiquitination and the proteasomal pathway. The presence of cysteine residues within Keap1 serves as a primary sensor of stress signals, which, under oxidative stress, lead to conformational alterations in Keap1. As a result, the ubiquitination of Nrf2 is inhibited, facilitating escape from Keap1-mediated degradation [40,41,42]. This leads to the translocation of Nrf2 into the nucleus, where it binds with the antioxidant-response element present in the promoter region of genes encoding antioxidant enzymes [43,44]. More than 250 Nrf2-targeted genes, including NAD(P)H: quinone oxidoreductase-1 (NQO1), heme oxygenase-1 (HO-1), glutamate–cysteine ligase, glutathione S-transferase, glutathione peroxidase, catalase, superoxide dismutase, thioredoxin, and UDP-glucuronosyltransferase, are reported to be transcriptionally regulated by Nrf2 [45]. Therefore, activation of Keap1–Nrf2 signaling is predicted to drive robust expression of these genes, thereby protecting cells from a variety of insults, including reactive toxicants, proinflammatory factors, apoptosis, and carcinogenesis [40,41].

In RPE cells, Nrf2 activation is essential because the retina is highly exposed to light, oxygen, and lipid-derived oxidants. Activation of Nrf2 is linked with the transcription of various antioxidant genes that help to maintain redox homeostasis and suppress oxidative damage [46,47]. Review articles focusing on retinal Nrf2 biology highlight the protective role of Nrf2 activation in retinal disease models, while the impairment of the Nrf2 pathway may aid in retinal aging and disease progression [46,48,49].

Studies demonstrate a crucial role of Nrf2 in retinal diseases. It has been found that patients with diabetic retinopathy show a decrease in Nrf2 DNA-binding activity in the retina [50], and a pivotal function of Nrf2 in the onset of AMD is documented [1,48]. In addition to this, age-related damage in the RPE, including accumulation of lipofuscin and drusen-like deposits and spontaneous choroidal neovascularization, is reported in Nrf2-knockout mice compared to wild-type controls [42,46,51]. Similarly, cigarette smoke exposure induced profound ultrastructural damage to the RPE–Bruch membrane complex in Nrf2-knockout mice, characterized by large cytoplasmic vacuoles and loss and dilation of basolateral infoldings [52]. Wu et al. showed that overexpression of Nrf2 in the RPE preserved the RPE morphology and survival in retinal degeneration mouse models. RPE-specific overexpression of Nrf2 protected the RPE cells and preserved the cone photoreceptor layer along with intact apical microvillus structure. Moreover, the transcriptomic analysis revealed that Nrf2 activated multiple antioxidant pathways and reversed the decline observed in the glutathione pathway in the control group [53]. Hence, the integration of oxidative stress with retinal deterioration and Nrf2-mediated antioxidant responses is motivating researchers to work towards identifying novel Nrf2 activators for the prevention and therapy of retinal damage [42,46,49,54].

### 4.2. NF-κB Signaling and Its Role in Retinal Inflammation

Inflammation is normally a protective mechanism, but it can trigger retinal damage when poorly regulated or reaches a chronic stage. In AMD, persistent inflammation can lead to RPE dysfunction and cell death. The pathogenesis of AMD, often driven by chronic inflammation, is fueled by activation of the NF-κB signaling pathway, which regulates the production of pro-inflammatory cytokines, chemokines, and angiogenic factors such as VEGF [55]. The oxidative stress and NF-κB pathways show a bidirectional relationship. Increased production of ROS can activate NF-κB signaling, and NF-κB-driven inflammation can lead to the generation of ROS through the induction of enzymes iNOS and COX-2 [55,56]. Blue light-induced photooxidation of A2E in RPE cells triggers NF-κB signaling and raises HMOX1 expression, thereby demonstrating NF-κB activation in an A2E-related photooxidative paradigm [57].

High-temperature requirement A serine peptidase 1 (HTRA1) is associated with inflammatory signaling in RPE cells. Pan et al. reported that lipopolysaccharide (LPS) activates NF-κB signaling, which in turn regulates HTRA1 expression in human retinal pigment epithelial cells. Thus, this study supports a role for NF-κB signaling upstream of HTRA1 expression rather than demonstrating that HTRA1 directly regulates NF-κB [58]. Treatment of ARPE-19 cells with a PI3K inhibitor abrogated the nuclear translocation of NF-κB and reduced the expression of inflammatory markers induced by hyperglycemia, indicating PI3K/Akt signaling-mediated regulation of NF-κB activity [59]. An increasing amount of data suggests that chemokine signaling has a role in the pathophysiology of AMD and retinal inflammation. In AMD, inflammatory cell recruitment, retinal degeneration, and choroidal neovascularization have all been linked to the CCL2/CCR2 and CX3CL1/CX3CR1 chemokine axes [60]. Besides this, the inflammatory activity of NF-κB in retinal degeneration is attributed to the high-mobility group box 2 (HMGB2), a pro-inflammatory factor, and damage-associated molecular patterns (DAMPs). HMGB2 translocates from the nucleus to the cytoplasm in photoreceptors and is released into the extracellular space upon oxidative stress. HMGB2 exerts its detrimental effect on photoreceptors via down-regulating Nrf2/HO-1 and activating NF-κB/NLRP3 signaling pathways [61].

### 4.3. Crosstalk of Nrf2 and NF-κB in Retinal Oxidative Stress and Inflammation

The pathophysiology of AMD is mostly driven by oxidative stress, which primarily affects RPE cells [51,62,63]. A deficit in Nrf2, a crucial regulator of retinal antioxidant defense, raises oxidative stress and causes progressive RPE degradation and AMD-like disease [51]. RPE cells’ capacity to trigger antioxidant genes including HO-1, NQO1, and GCLM under oxidative stress is diminished by an aging-associated reduction in Nrf2 signaling, which increases ROS buildup and heightens susceptibility to AMD [62]. In contrast, oxidative stress, mitochondrial dysfunction, A2E accumulation, and drusen-associated cholesterol crystals activate NF-κB in RPE cells, causing expression of pro-inflammatory cytokines like TNF-α, IL-1β, IL-6, and IL-8. This promotes chronic inflammation and the advancement of AMD [63]. NF-κB signaling is adversely modulated by Nrf2 activation via a variety of regulatory mechanisms. Nrf2-induced HO-1 limits NF-κB nuclear translocation, thereby lowering the production of pro-inflammatory mediators and restricting oxidative stress-induced inflammation. Separately, Keap1-mediated degradation of IKKβ decreases NF-κB activation [64,65]. Additionally, oxidative stress and inflammatory signaling in RPE cells are made worse by mitochondrial ROS generation and poor autophagic clearance [32,66]. Further experimental research has demonstrated the antagonistic interaction between these two signaling pathways by demonstrating that Nrf2 depletion increases NF-κB transcriptional activity and inflammatory gene expression [67,68]. Significantly, oxidative stress and inflammation are increased when Nrf2 signaling is disrupted, and pro-inflammatory responses are encouraged when NF-κB activation persists. This creates a self-amplifying cycle that speeds up retinal degeneration in AMD [51,63].

## 5. Marine Carotenoids as Retinal Protective Agents

Of the 36 recognized animal phyla, 34 are found in the marine environment, which makes up about 70% of the Earth’s surface [69]. This biodiversity remains largely unexplored and offers substantial biotechnological potential. Marine-derived carotenoids are distinct bioactive secondary metabolites obtained from seaweeds and microalgae. Owing to their strong anti-inflammatory, cytoprotective, and antioxidant properties, they hold considerable promise and enormous potential in the prevention and treatment of oxidative stress-related diseases [70,71]. In recent years, marine resources have garnered considerable interest in the therapy of retinal diseases [72]. The use of marine resources as food is backed by scientific evidence in several studies highlighting their beneficial effects in mitigating retinal diseases [73]. Several studies have unveiled marine resources with strong potential for preventing or slowing retinal diseases by exerting anti-inflammatory, antioxidant, vasoprotective, and cytoprotective activities or by ameliorating retinal function [72].

In addition, research on carotenoid management within the RPE itself is ongoing: a recent study found that Aster-B-mediated non-vesicular carotenoid transport is a determinant of oxidative stress responses in ARPE-19 cells, highlighting the fact that intracellular carotenoid distribution, rather than just antioxidant capacity, influences how RPE cells react to oxidative challenge [74].

Astaxanthin, a marine-derived xanthophyll carotenoid, is produced by a limited number of plants, bacteria, fungi, and algae, predominantly microalgae. The microalga *Haematococcus pluvialis* is considered one of the richest natural sources of astaxanthin, where it accumulates under stressful environmental conditions [75]. Astaxanthin is particularly effective at protecting phosphatidylcholine liposomes against lipid peroxidation and shows strong antioxidant activity at pHs 7.4 and 8 [76]. Astaxanthin exhibits antioxidant and retinal protective effects against ischemia/reperfusion-induced damage, suggesting its potential as a neuroprotective agent in retinal disorders [77]. However, due to poor public awareness, high production costs, and limited large-scale availability, its commercial and clinical applicability is still restricted [78]. Its anti-inflammatory and antioxidant activities may also contribute to its potential in oxidative stress-related disorders [79].

Fucoxanthin is a marine-derived orange xanthophyll carotenoid found mainly in brown algae and diatoms such as *Undaria pinnatifida*, *Laminaria japonica*, *Phaeodactylum tricornutum* and *Cylindrotheca closterium* [80,81]. It exhibits antioxidant, anti-inflammatory, and cytoprotective properties [80,82,83,84,85] and enhances the Nrf2/ARE signaling pathway and antioxidant enzymes such as HO-1 and NQO1 [86]. Fucoxanthin also protects RPE and photoreceptor cells by filtering blue light, reducing ROS, MDA, and RPE senescence, improving mitochondrial and phagocytic function, and decreasing VEGF [87,88].

Zeaxanthin is a xanthophyll carotenoid concentrated in the central retina, where it contributes to the macular pigment and protects against photooxidative stress and lipid peroxidation through blue-light screening and antioxidant activity [89,90,91]. Zeaxanthin is also produced by marine microorganisms isolated from saltwater, such as *Gramella planctonica* and *Aquibacter zeaxanthinifaciens* and is obtained from dietary sources such as corn [92,93,94]. Direct evidence for zeaxanthin in an A2E-specific model is available. Bian et al. supplemented A2E-laden ARPE-19 cells with zeaxanthin before blue-light exposure and reported reduced photooxidation-induced proteasome inactivation and attenuated changes in MCP-1, IL-8, and CFH expression [95]. However, this evidence is limited to A2E and has not been extended to other bisretinoids [89,90].

Lutein is a C40 oxygenated carotenoid structurally related to zeaxanthin [96,97] and is naturally found in fruits, vegetables, flowers, and other plant sources [94]. Microalgae of the *Chlorella* genus are important commercial sources of lutein due to their rapid growth and high pigment content [98,99,100,101]. Lutein is widely used as a nutraceutical due to its antioxidant and photoprotective properties and preferential accumulation in the macula and lens [97]. Age-related macular degeneration and cataract are among the major causes of blindness and vision impairment worldwide [102]. Reduced lutein accumulation has been associated with age-related macular degeneration and cataracts [103,104]. Its health-promoting properties also support its use as a nutraceutical and natural food colorant [105].

These xanthophyll carotenoids share polyene-rich structures that confer strong antioxidant and anti-inflammatory activity [70,106]. The sources, key biological activities, molecular targets, and relevance to retinal disease of these carotenoids are summarized in Table 1.

Although lutein and zeaxanthin have more direct experimental evidence in A2E-loaded RPE models than astaxanthin (Section 7), astaxanthin remains the primary focus of this review for three reasons. First, astaxanthin has been reported to exhibit high singlet-oxygen quenching capacity [107] and a membrane-spanning configuration facilitated by its polar end groups [96,108], properties that are mechanistically relevant to the singlet-oxygen chemistry underlying A2E photoexcitation described in Section 3. Second, astaxanthin has been reported to modulate Nrf2, NF-κB, and PI3K/Akt-related signaling in ARPE-19 cells (refs. [17,59] providing a broad mechanistic framework for its potential activity (Section 6). Third, the absence of direct testing of astaxanthin in an A2E-specific model constitutes a clearly defined evidence gap and provides a strong rationale for further investigation in a model relevant to bisretinoid pathology. The discussion of fucoxanthin, lutein, and zeaxanthin is therefore limited to information necessary to establish this rationale and contextualize the evidence gap discussed in Section 7.

**Table 1:** Carotenoids relevant to retinal protection and their principal natural sources, with marine and microalgal sources emphasized.

Carotenoids	Natural Sources	Key Biological Activities	Molecular Targets/Pathways	Effects on Retinal Diseases	Key Significance	Ref.
Astaxanthin	*Haematococcus pluvialis (H. pluvialis)*	Strong antioxidant, anti-inflammatory, anti-apoptotic	Nrf2/HO-1, NF-κB inhibition, ROS scavenging	Protects RPE from oxidative and light-induced damage (H_2_O_2_, general photooxidative stress) ^1^	Strong singlet-oxygen quenching capacity	[14,17]
Fucoxanthin	Brown algae (*Undaria pinnatifida, Laminaria japonica); diatoms (Phaeodactylum tricornutum, Cylindrotheca closterium*)	Antioxidant, anti-inflammatory, anticancer, antihypertensive, anti-obesity, photoprotective and neuroprotective	Nrf2 activation	Reduces oxidative stress in retinal cells	Emerging marine carotenoid for AMD	[82,83,84,85,109,110,111]
Zeaxanthin	Marine bacteria (*Gramella planctonica, Aquibacter zeaxanthinifaciens*), Corn	Blue light filter, antioxidant, anti-inflammatory and structural actions in neural tissue	ROS quenching, membrane stabilization	Tested in A2E-laden RPE cells exposed to blue light; reduced photooxidation-induced proteasome inactivation and attenuated changes in MCP-1, IL-8, and CFH expression, and it also supports macular protection and visual function	Accumulates in macula (macular pigment)	[89,92,93,94,95,112]
Lutein	Microalgae *(Chlorella protothecoides*, *Scenedesmus almeriensis*), Green leafy vegetables	Antioxidant, anti-inflammatory	Nrf2, NF-κB inhibition	Tested in A2E-laden RPE cells exposed to blue light; reduced photooxidative damage and modulated inflammation-related gene expression, and it also supports protection against retinal damage and AMD	Works synergistically with zeaxanthin	[89,95,98,99,100,112,113,114]
β-carotene	Algae (*Dunaliella salina, Chlorella zofingiensis),* Carrots	Provitamin A, antioxidant	Retinoid cycle involvement	Supports vision but less effective in AMD protection	Provitamin A carotenoid cleaved by BCO1 to retinal	[113,115,116,117]

^1^ Astaxanthin has not been tested in an A2E-specific model. Available retinal studies primarily involve general oxidative or photooxidative stress models.

## 6. Astaxanthin as a Key Modulator of Nrf2 and NF-κB Crosstalk

**Figure 2:**
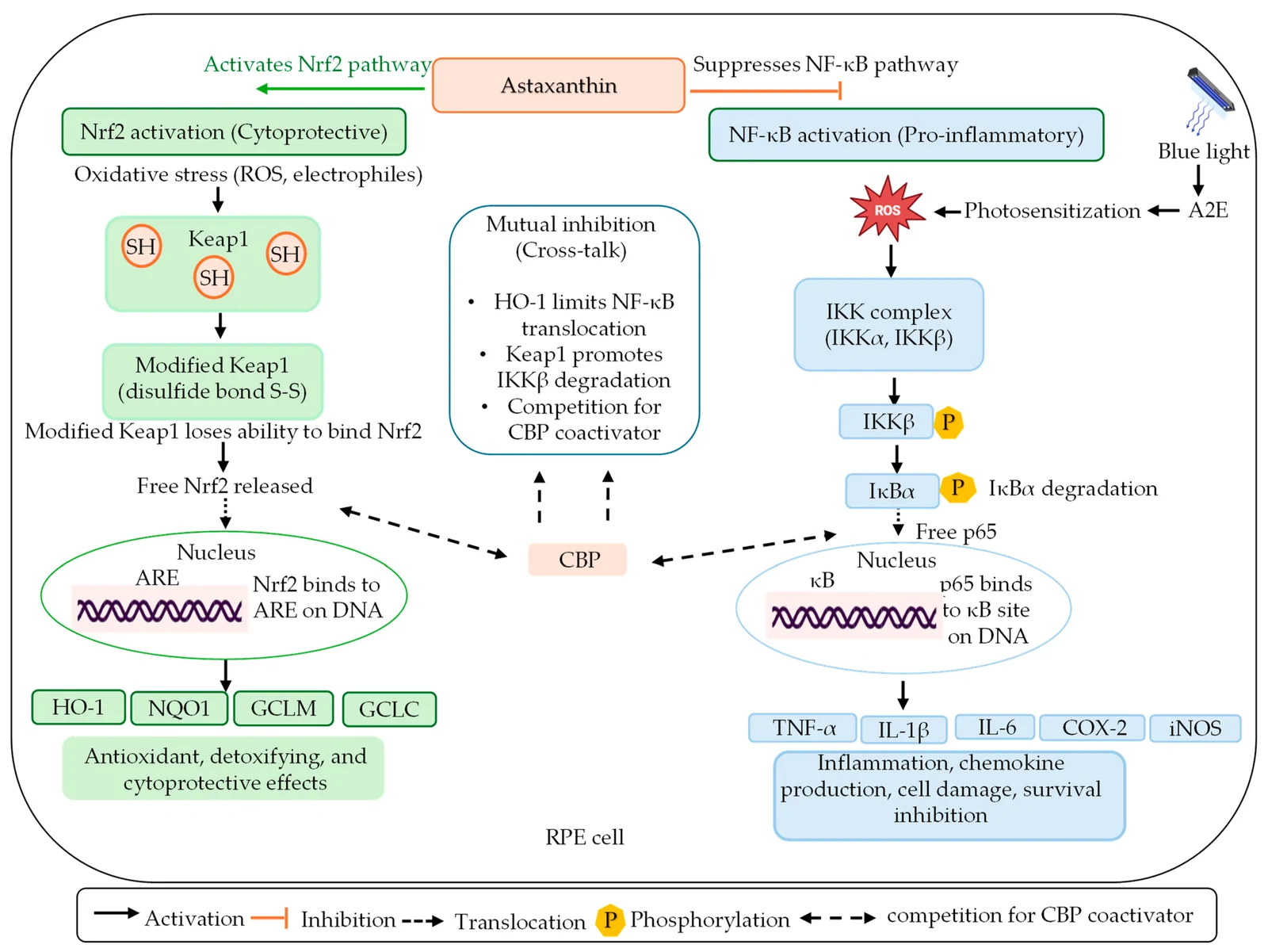
Nrf2 and NF-κB crosstalk and the action points of astaxanthin. In RPE cells, blue-light excitation of A2E generates ROS through photosensitization, which initiates NF-κB activation (IKK → IκBα degradation→p65 nuclear translocation), driving pro-inflammatory genes (TNF-α, IL-1β, IL-6, COX-2, iNOS); astaxanthin suppresses this pathway. Oxidative stress also modifies Keap1, releasing Nrf2 to activate ARE-driven cytoprotective genes (HO-1, NQO1, GCLM, GCLC); astaxanthin activates this pathway. GCLC upregulation has been reported in ARPE-19 cells, whereas GCLC expression was not reported in the 661W photoreceptor cell study. The two pathways mutually inhibit each other via HO-1, Keap1, and competition for the CBP coactivator. The underlying photochemistry is detailed in Figure 1. Legend: Black solid arrow, activation; orange flat-ended line, inhibition; black dashed arrow, translocation; orange circled “P,” phosphorylation; green solid arrow, astaxanthin-mediated activation of the Nrf2 pathway; black double-headed dashed arrow, competition for CBP coactivator. Abbreviations: IKK, inhibitor of nuclear factor kappa B kinase complex; SH, sulfhydryl (thiol) group; S–S, disulfide bond. ARE, antioxidant response element; CBP, CREB-binding protein; COX-2, cyclooxygenase-2; GCLM, glutamate–cysteine ligase modifier subunit; GCLC, glutamate–cysteine ligase catalytic subunit; HO-1, heme oxygenase-1; IκBα, inhibitor of nuclear factor kappa B alpha; IKKα, inhibitor of nuclear factor kappa B kinase alpha; IKKβ, inhibitor of nuclear factor kappa B kinase beta; IL-1β, interleukin-1 beta; IL-6, interleukin-6; iNOS, inducible nitric oxide synthase; Keap1, Kelch-like ECH-associated protein 1; NF-κB, nuclear factor kappa B; NQO1, NAD(P)H: quinone oxidoreductase 1; Nrf2, nuclear factor erythroid 2-related factor 2; ROS, reactive oxygen species; RPE, retinal pigment epithelium; TNF-α, tumor necrosis factor-alpha.

Marine carotenoids appear to be potential bioactive substances for the treatment and prevention of several illnesses, according to mounting data [118,119]. These substances work by controlling many cellular signaling pathways, especially the Nrf2 and NF-κB signaling cascades [120]. Astaxanthin has been shown to have a crucial role in modifying these transcription factors over the past 15 years, contributing to a variety of positive health effects [121,122]. The Nrf2/NF-κB crosstalk and astaxanthin’s points of action are illustrated in Figure 2. Where the evidence for a given mechanism derives from non-retinal systems, such as acute lung injury models, gastric epithelial cells, macrophages, or cell-free liposomes, it is cited here as evidence of mechanistic plausibility only. The boundary applied to such extrapolation is defined in Section 2.2, and the corresponding models are graded as non-retinal in the evidence table in Section 7. 

### 6.1. Chemical Structure and Biological Properties

**Figure 3:**
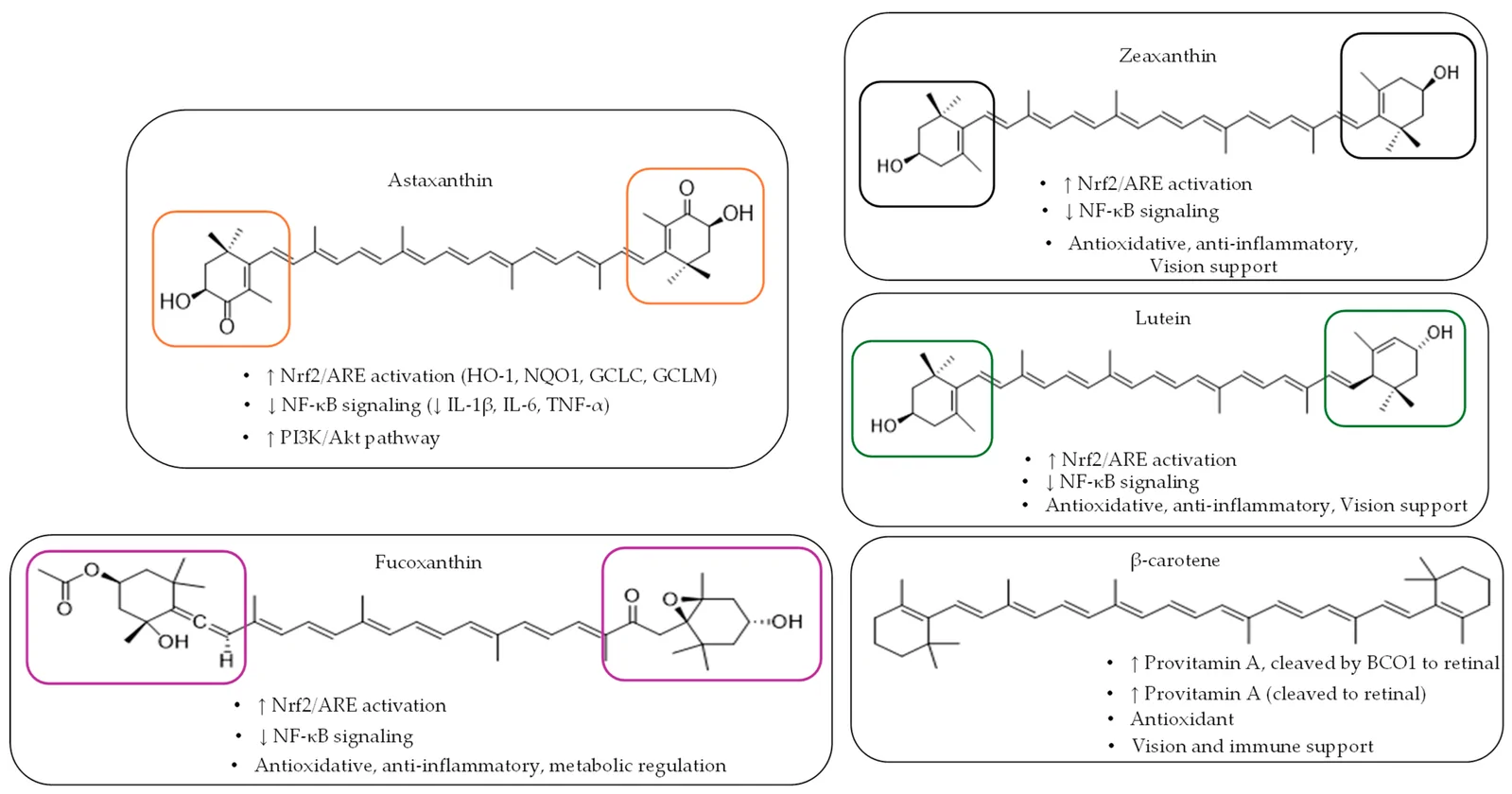
Structural and pathway comparison of selected carotenoids with emphasis on astaxanthin. Chemical structures of astaxanthin, fucoxanthin, lutein, zeaxanthin, and β-carotene are shown together with their principal signaling routes. Functional end-groups are indicated in colored boxes. Upward (↑) and downward (↓) arrows denote increased/activated and decreased/inhibited activity or signaling, respectively. Astaxanthin exerts strong antioxidative and anti-inflammatory effects primarily by activating Nrf2/ARE signaling and suppressing NF-κB signaling. It activates the PI3K/Akt pathway and modulates downstream targets including HO-1, NQO1, GCLM, GCLC, as well as the cytokines IL-1β, IL-6, and TNF-α. The other carotenoids are included for comparative context only: fucoxanthin, lutein, and zeaxanthin also act via Nrf2/ARE and NF-κB, while β-carotene acts through a distinct route as a provitamin A carotenoid cleaved by BCO1 to retinal, the precursor of retinoic acid, supporting immune, ocular, and antioxidant functions. **Abbreviations:** BCO1, β-carotene-15,15′-oxygenase 1; ARE, antioxidant response element; GCLC, glutamate–cysteine ligase catalytic subunit; GCLM, glutamate–cysteine ligase modifier subunit; HO-1, heme oxygenase-1; IL-1β, interleukin-1 beta; IL-6, interleukin-6; NF-κB, nuclear factor kappa B; NQO1, NAD(P)H: quinone oxidoreductase 1; Nrf2, nuclear factor erythroid 2-related factor 2; PI3K, phosphoinositide 3-kinase; Akt, protein kinase B; TNF-α, tumor necrosis factor-alpha.

Astaxanthin is a marine-derived xanthophyll carotenoid characterized by a unique molecular structure containing conjugated double bonds and terminal hydroxyl and keto groups, which confer exceptional antioxidant activity. By efficiently scavenging reactive oxygen species (ROS), quenching singlet oxygen, and inhibiting lipid peroxidation within biological membranes, astaxanthin exhibits potent cytoprotective properties that often surpass those of many conventional antioxidants [123]. A lipophilic xanthophyll carotenoid (C_40_H_52_O_4_), astaxanthin has polar ionone rings with hydroxyl and keto groups and a conjugated polyene chain. Its unique shape facilitates membrane bridging and adds to its potent biological and antioxidant properties [108]. Astaxanthin’s basic structural characteristic is a polyene chain composed of conjugated C=C bonds, as in other carotenoids. Astaxanthin is a 3,3′-dihydroxy*-β,β-*carotene-4,4′-dione according to its molecular structure. It has two equivalent chiral centers at the 3 and 3′ positions of the β-ionone ring, with a hydroxyl group at both ends. Astaxanthin’s 3,3′ asymmetric carbons enable the formation of three potential optical isomers with an all-*trans* chain configuration: 3*R*,3′*R*, 3*S*,3′*S*, and 3*R*,3′*S* [124]. Astaxanthin exhibits a wide range of biological activities, such as anti-inflammatory, antidiabetic, cardioprotective, neuroprotective, immunomodulatory, and anticancer effects. It also exhibits strong free radical-scavenging efficiency compared with several other carotenoids and traditional antioxidants. Furthermore, astaxanthin enhances endogenous antioxidant defense systems, reduces inflammatory mediators, and modulates oxidative stress-related signaling cascades. By shielding cellular macromolecules from oxidative damage, it holds the potential therapeutic potential for chronic oxidative stress-associated disorders [14]. The structural features and main signaling routes of astaxanthin and other marine carotenoids are compared in Figure 3.

### 6.2. Activation of Nrf2-Dependent Antioxidant Pathways

Astaxanthin engages the Keap1–Nrf2/ARE signaling pathway detailed in Section 4.1 [40,41]. Growing evidence indicates that astaxanthin enhances this pathway by increasing Nrf2 activation and the production of downstream antioxidant enzymes, thereby reducing oxidative damage and improving retinal cell survival [17,125,126].

In retinal photoreceptor cells, astaxanthin and its analogues activate the Nrf2 signaling pathway, increase Nrf2 nuclear translocation, and upregulate downstream antioxidant genes, including HO-1, NQO1, and GCLM. Astaxanthin and its analogues reduced the production of intracellular ROS in 661 W photoreceptor cells and provided protection against oxidative damage and cell death brought on by light. Notably, Nrf2 silencing eliminated these cytoprotective effects, suggesting that endogenous antioxidant defense mechanisms and Nrf2 activation are crucial for astaxanthin-mediated retinal protection [125].

Similarly, in acute glaucoma models, astaxanthin inhibits retinal ganglion cell death and maintains retinal integrity by activating the Nrf2/HO-1 pathway. Astaxanthin reduces ischemia/reperfusion-induced retinal damage via regulating apoptotic markers like Bax and Bcl-2 and boosting antioxidant defense signaling, while Nrf2 knockdown reduces its neuroprotective benefits [126].

Astaxanthin enhances the expression of HO-1, NQO1, GCLM, and GCLC at both the mRNA and protein levels in ARPE-19 cells via activating the Nrf2/ARE pathway, with its protective effects also associated with PI3K/Akt signaling [17]. Collectively, these studies indicate that Nrf2 activation and subsequent induction of antioxidant enzymes are important components of astaxanthin-mediated protection against oxidative stress in retinal cells [17,125,126,127].

### 6.3. Inhibition of NF-κB-Mediated Inflammatory Responses

A highly controlled set of immunological reactions, inflammation serves as a defense against damaging stimuli and is essential for starting tissue repair and re-establishing homeostasis after injury [128]. NF-κB proteins are a family of transcription factors that are essential for controlling cellular growth, proliferation, apoptosis, inflammation, and immunological function. Many target genes, such as cytokines, chemokines, immunoreceptors, cell-adhesion molecules, stress-responsive genes, apoptosis regulators, growth factors, and other transcription factors involved in various physiological and pathological processes, are regulated by NF-κB signaling [129]. However, severe or poorly controlled inflammation can be harmful to the host, causing damage to surrounding tissues and cells [130]. Astaxanthin has been shown to have a variety of biological effects and is considered one of the most potent natural antioxidants. Astaxanthin has systemic anti-inflammatory and neuroprotective effects by slowing the development of diseases of the central nervous system and reducing inflammation in important organs. Suppression of NF-κB signaling, which in turn prevents the generation of downstream inflammatory cytokines including IL-1β, IL-6, and TNF-α, is a major mechanism by which these protective effects are mediated [131,132,133,134]. By altering the MAPK and nuclear factor signaling pathways, astaxanthin has strong anti-inflammatory actions that lower TNF-α and IL-6 levels and lessen acute lung injury [135]. Furthermore, astaxanthin demonstrates its anti-inflammatory potential by reducing the expression of interleukin-8 (IL-8) in infected gastric epithelial cells [136]. These results from non-retinal models confirm astaxanthin’s wider anti-inflammatory properties. Crucially, research unique to the retina also suggests that astaxanthin can regulate oxidative and inflammatory reactions in diabetic retinal tissue and ARPE-19 cells [59]. Nevertheless, there is still a lack of direct mechanistic evidence describing astaxanthin-mediated Nrf2–NF-κB crosstalk in RPE cells under photooxidative or bisretinoid-induced stress, underscoring the need for additional RPE-specific research to ascertain whether astaxanthin has comparable effects under retinal oxidative and photooxidative stress.

### 6.4. Astaxanthin Regulates the Functional Interaction Between Nrf2 and NF-κB

The general crosstalk between Nrf2 and NF-κB in retinal oxidative stress and inflammation is described in Section 4.3. This section focuses on the evidence for astaxanthin-mediated modulation of Nrf2 and NF-κB signaling and its possible significance to retinal protection, building on this well-established mechanism. The Nrf2 and NF-κB signaling pathways reciprocally interact to enhance the pathogenic processes of oxidative stress and inflammation [137]. Nrf2 is a key transcriptional regulator that ensures redox homeostasis by coordinating the expression of genes involved in cellular protection, anti-inflammatory action, and antioxidant defense, whereas pro-inflammatory stimuli like TNF-α, IL-1β, and lipopolysaccharide (LPS) activate NF-κB, a crucial transcription factor that controls immunological and inflammatory responses. This triggers the transcription of genes linked to inflammation [137,138]. Crucially, persistent NF-κB activation may inhibit Nrf2-mediated transcription through competition for transcriptional co-activators like CBP, upsetting cellular redox balance, while Nrf2 activation can suppress NF-κB signaling by lowering intracellular ROS levels and boosting antioxidant defenses [137]. According to recent research, astaxanthin’s cytoprotective effects are achieved by coordinated stimulation of Nrf2 signaling and repression of NF-κB-driven inflammatory responses, which eventually modifies the crosstalk between these pathways [139] (Figure 2).

AMD and RPE dysfunction have been linked to the interaction between the Nrf2 and NF-κB signaling pathways. By stimulating the expression of antioxidant response element (ARE)-driven genes, such as heme oxygenase-1 (HO-1), NAD(P)H quinone oxidoreductase-1 (NQO1), and glutamate–cysteine ligase (GCL), Nrf2 functions as a master regulator of the cellular antioxidant defense system under normal physiological conditions. Nrf2 protects intracellular redox equilibrium and increases RPE cells’ resilience to oxidative stress by coordinating the production of these cytoprotective enzymes [53,140]. Studies demonstrate that genetic disruption of Nrf2 causes AMD-like pathological features, such as RPE degeneration, lipofuscin accumulation, drusen-like deposits, spontaneous choroidal neovascularization, and increased sub-RPE inflammatory protein deposition, further highlighting the crucial role of Nrf2 in maintaining retinal homeostasis [51]. Additional evidence from experimental and clinical studies suggests that astaxanthin mitigates oxidative stress by decreasing biomarkers of lipid and protein oxidation while simultaneously enhancing endogenous antioxidant defenses, including glutathione levels and the activities of superoxide dismutase and catalase [140,141,142].

In addition to its antioxidant properties, astaxanthin suppresses the production of downstream inflammatory mediators, including COX-2 and iNOS, and inhibits NF-κB signaling [143]. Experimental CNV studies have demonstrated that astaxanthin attenuates NF-κB signaling, suppresses inflammatory mediator production, and inhibits both macrophage recruitment and pathological neovascularization, although there is still little direct mechanistic evidence connecting astaxanthin to Nrf2–NF-κB crosstalk in AMD. Together, these results imply that astaxanthin may protect the retina by controlling inflammatory pathways, while more research is needed to determine how it affects coordinated Nrf2–NF-κB signaling [144].

According to Farruggia et al., astaxanthin lowers intracellular ROS levels while blocking NF-κB p65 nuclear translocation through both Nrf2-dependent and Nrf2-independent mechanisms, supporting the reciprocal regulation of oxidative stress and inflammatory signaling. This suggests that astaxanthin may simultaneously modulate several cytoprotective pathways [139]. All these results suggest that astaxanthin may aid in re-establishing the equilibrium between NF-κB-driven inflammation and Nrf2-mediated antioxidant responses.

## 7. Astaxanthin in Bisretinoid-Induced Retinal Damage

A2E accumulation, which increases with age and is markedly accelerated by ABCA4 loss of function, and its subsequent photooxidation in RPE cells are associated with oxidative stress, mitochondrial dysfunction, lysosomal impairment, and persistent inflammatory signaling, all of which contribute to retinal degeneration and the processes implicated in AMD [20,28,29], as explained in Section 3. Although lutein and zeaxanthin have been tested in A2E-containing ARPE-19 cells exposed to blue light and were shown to attenuate photooxidation-induced proteasome inactivation and changes in inflammation-related genes, astaxanthin has not been tested in an A2E-specific model [95]. Most investigations of astaxanthin have instead assessed models of oxidative stress caused by hydrogen peroxide, general light-induced retinal injury, or other non-A2E oxidative challenges. Determining whether astaxanthin can directly reduce A2E-induced photooxidative damage is therefore still a top research topic. The proposed mechanism of A2E-mediated photooxidative damage and the points at which astaxanthin is hypothesized to intervene are summarized in Figure 1.

Upon blue-light excitation, bisretinoids can generate reactive oxygen species (ROS), including singlet oxygen, thereby contributing to photooxidative damage. Carotenoids’ capacity to scavenge ROS may shield retinal tissues from oxidative stress linked to lipofuscin and lessen oxidative damage [145]. One common in vitro model of bisretinoid-induced retinal damage is A2E-loaded ARPE-19 cells exposed to blue light (430 nm). In this model, blue-light excitation of A2E causes ROS-mediated oxidative stress, which activates NF-κB and increases the expression of HMOX1 (HO-1) via MLL1-dependent chromatin remodeling and NF-κB (RELA) recruitment. The therapeutic significance of this oxidative stress-responsive signaling pathway is further highlighted by the finding of increased HMOX1 levels in retinal tissues from individuals with late-stage AMD [57]. Otsuka et al. showed that supplementing mice with astaxanthin reduced retinal damage brought on by prolonged exposure to light. Treatment reduced oxidative DNA damage, as seen by lower levels of 8-hydroxy-2′ deoxyguanosine (8-OHdG), while preserving retinal function, as demonstrated by enhanced electroretinogram (ERG) responses and maintenance of outer nuclear layer (ONL) thickness [146]. The results indicate that astaxanthin may shield retinal cells from photooxidative stress, a crucial mechanism behind bisretinoid-induced retinal damage, even though this study did not specifically assess A2E-mediated toxicity.

Astaxanthin decreased intracellular ROS levels, enhanced cell survival, and reduced apoptosis in ARPE-19 cells after exposure to H_2_O_2_ through PI3K/Akt-associated Nrf2 activation and upregulation of antioxidant enzymes, such as HO-1, NQO1, GCLC, and GCLM [17]. However, this study offers evidence for a broad antioxidant mechanism rather than direct proof of protection against A2E-mediated damage because it employed H_2_O_2_ instead of A2E or blue-light-induced bisretinoid photooxidation. Other xanthophyll carotenoids have been shown to have similar protective properties. Specifically, by triggering the Nrf2 signaling pathway, fucoxanthin has been demonstrated to reduce oxidative stress caused by visible light and restore phagocytic activity in RPE cells, highlighting the crucial role of Nrf2-mediated antioxidant defenses in retinal protection [110]. In mitochondrial-mimetic liposome systems, astaxanthin has been shown to reduce oxidative and nitrative lipid peroxidation, minimizing membrane damage and blocking pro-apoptotic oxidative pathways mediated by cytochrome c. These findings demonstrate its potent antioxidant qualities and point to a protective function in preserving cellular integrity in the face of oxidative stress [76].

Astaxanthin has not been tested against A2E-induced retinal damage, but photooxidative models based on all-*trans*-retinal (at-RAL) provide partial mechanistic insight. In at-RAL-exposed ARPE-19 cells, blue light generates singlet oxygen, and astaxanthin suppresses the resulting oxidative damage and apoptosis by scavenging singlet oxygen directly [107]. The relevance of this finding to A2E pathology is limited in three respects. First, at-RAL is an enzymatically regulated visual cycle intermediate that is cleared by ABCA4-mediated transport and retinol dehydrogenase 8 (RDH8) and retinol dehydrogenase 12 (RDH12)-dependent reduction, whereas A2E resists enzymatic degradation and accumulates in RPE lysosomes. Exogenous at-RAL loading therefore models acute failure of retinoid clearance rather than chronic bisretinoid accumulation. Second, at-RAL is the precursor of A2E rather than a parallel photosensitizer, and the two species differ in subcellular distribution and in photochemistry. The self-sensitized photooxidation and subsequent photocleavage that generate the aldehyde and dicarbonyl adducts central to A2E toxicity have no counterpart in the at-RAL system. Third, in A2E-laden cells astaxanthin must compete with the polyene chain of A2E itself for singlet oxygen addition, and singlet-oxygen scavenging in an at-RAL system does not establish that astaxanthin can intercept singlet oxygen before it reacts with A2E. In addition, the at-RAL study used 450 nm blue light, whereas A2E absorbs near 430 nm. The at-RAL model is therefore the closest available mechanistic proxy, but confirmation in A2E-specific models remains necessary [107].

The boundaries of the indirect comparison with the macular xanthophylls are made clear by a horizontal comparison of experimental circumstances. The only A2E-specific carotenoid experiment identified in our review involved loading ARPE-19 cells with 10 µM A2E for 10 days, followed by treatment with 10 µM lutein or zeaxanthin for 3 days in A2E-free medium, and subsequent irradiation for 10 min at 430 ± 20 nm and 2.62 mW/cm^2^ [95]. While another RPE study utilized 200 µM H2O_2_ with 10 and 20 µM astaxanthin [17], the closest astaxanthin experiment employed 2 µM at-RAL with 0–2 µM astaxanthin pretreatment and 450 nm, 7 mW/cm^2^ and 10 min irradiation [107]. Relative potency cannot be determined due to differences in the photosensitizer, loading duration, irradiance, wavelength, and carotenoid concentrations. A physicochemical and mechanistic explanation for astaxanthin includes singlet-oxygen quenching and documented effects on Nrf2, NF-κB, and PI3K/Akt [17,59,107], whereas lutein and zeaxanthin are known macular xanthophylls with direct empirical proof in A2E-laden cells [95]. A head-to-head experiment within a single A2E paradigm is necessary to determine whether astaxanthin is more or less efficient than lutein or zeaxanthin against A2E photooxidation.

The data supporting astaxanthin’s protective effect against A2E-mediated damage are indirect and vary in methodology among the studies examined in this section. The animal studies in Table 2 (Supplementary Table S1) also have methodological limitations, including unclear or absent reporting of randomization, allocation concealment, blinded outcome assessment, and a priori sample-size estimation, which limits the strength of the in vivo evidence. The most compelling functional evidence is found in an in vivo mouse model where dietary astaxanthin supplementation decreased oxidative DNA damage and maintained retinal structure and ERG responses under extended light exposure [146]; however, because this model used general light-induced injury instead of A2E-loaded cells, it is unable to establish a mechanism specific to bisretinoids. The remaining evidence comes from in vitro ARPE-19 models using different oxidative challenges: exposure to H_2_O_2_ [17], mitochondrial-mimetic liposome peroxidation [76], and, most importantly, all-*trans*-retinal photooxidation with blue light, where astaxanthin directly scavenged singlet oxygen and decreased apoptosis [107]. Because blue-light excitation of A2E generates singlet oxygen, the at-RAL model [107] shares this feature with A2E photooxidation but lacks the bisretinoid-specific photooxidation and photodegradation steps; therefore, it remains a proxy rather than a direct test of A2E photooxidation. A validated A2E-loaded, blue-light-exposed RPE model, the system most directly relevant to bisretinoid pathology described in Section 3, does exist [57], but astaxanthin has not been tested within it. Similarly, dosing, duration of light exposure, and outcome measures (biochemical markers vs. functional ERG readouts vs. apoptosis assays) vary between studies, making it impossible to compare the protective magnitude quantitatively. Studies utilizing A2E-specific experimental systems with standardized doses and exposure methods will be necessary to determine whether astaxanthin acts directly on A2E photodegradation products [22] rather than only on generic oxidative stress pathways. Table 2 summarizes these models and their relevance to A2E-specific pathology. The endpoints for ferroptosis and mitochondrial fragmentation outlined in Section 3 [36,37] also expand the scope of what an initial astaxanthin test in this context should measure. Although astaxanthin partitions into mitochondrial membranes and suppresses phospholipid peroxidation in mitochondrial-mimetic systems [76], it has not been tested against either route. This makes the readouts for an A2E-specific experiment both reasonable and easily measurable. Similarly, a potential protective effect should ideally be confirmed in at least one polarized or primary RPE system, not just in ARPE-19 cells, because the A2E-induced phagolysosomal and cholesterol-handling defects described in Section 3 are reproducible across ARPE-19, D407, RPE-J, and primary bovine RPE cells [34,35]. Table 2 contains these extra models and endpoints.

## 8. Clinical Evidence and Bioavailability Constraints

### 8.1. Clinical Evidence

Although most of the evidence supporting the use of astaxanthin-containing carotenoid formulations in retinal diseases comes from experimental research, a small number of clinical trials indicate that these formulations may enhance visual function in patients with AMD. Astaxanthin-containing supplements have been linked to increases in visual acuity, contrast sensitivity, retinal function, and quality of life related to vision, indicating possible therapeutic advantages for retinal health [147,148]. A carotenoid supplement containing astaxanthin enhanced central retinal function in patients with nonadvanced AMD after 12 months of supplementation, according to a clinical trial by Parisi et al. [147]. The clinical potential of astaxanthin-containing formulations for retinal health is further supported by a Carotenoids in Age-Related Maculopathy Italian Study (CARMIS), randomized trial that found that supplementing patients with AMD with lutein, zeaxanthin, and astaxanthin for two years improved visual acuity, contrast sensitivity, and vision-related function [148]. Crucially, instead of two separate clinical trials, these reports reflect a single carotenoid combination trial. Rather than being used as a stand-alone treatment, astaxanthin was given as part of a formulation that also included lutein and zeaxanthin [147,148]. Therefore, the observed improvements in visual acuity, contrast sensitivity, and retinal function cannot be directly attributed to astaxanthin because the trial did not include an astaxanthin-only arm; synergistic, additive, or confounding effects of lutein and zeaxanthin cannot be ruled out. Astaxanthin monotherapy has not yet been tested against a recognized visual outcome in AMD or other retinal diseases in a sufficiently powered, placebo-controlled trial.

### 8.2. Bioavailability Constraints

Astaxanthin, a highly lipophilic xanthophyll carotenoid, has poor and variable oral bioavailability because of its limited solubility in aqueous environments. Naturally occurring astaxanthin is usually found in esterified (mono- and diester) form and needs to be hydrolyzed by cholesterol esterase to facilitate micellar integration and absorption [149]. Consequently, pharmacokinetic studies estimate that approximately 10–50% of an administered dose is absorbed [149]. Moreover, nanocarrier-based delivery systems (nanoemulsions, liposomes, solid lipid nanoparticles, and polymeric nanoparticles) have been developed specifically to improve astaxanthin’s dissolution and absorption in comparison to the free compound [150]. Cross-study comparison is made more difficult by the varied reporting of these formulation changes across research trials. However, despite improvements in systemic absorption, significant uncertainties remain regarding the tissue distribution of orally administered astaxanthin, particularly whether sufficient concentrations reach the retina or retinal pigment epithelium to produce pharmacological effects [149].

### 8.3. Retinal Exposure After Oral Dosing

In the context of systemic exposure following oral administration, astaxanthin doses exhibiting protective benefits in vitro can be taken into consideration. Astaxanthin at 10 and 20 µM was tested in ARPE-19 cells treated with H_2_O_2_ [17] and at 25 µM in mitochondrial-mimetic liposomes [76]. Additionally, astaxanthin has been demonstrated to shield at-RAL-exposed ARPE-19 cells against phototoxicity caused by blue light [107]. Oral exposure is significantly impacted by formulation and administration conditions, according to human pharmacokinetic studies. A mean elimination half-life of 15.9 ± 5.3 h and formulation-dependent plasma exposure were observed with a single 40 mg oral dosage [151]. A more recent investigation found that an oral dose of 8 mg resulted in plasma *C*_max_ values of 7.21 µg/mL for a micellar formulation and 3.86 µg/mL for native astaxanthin [152]. These plasma *C*max values are substantially higher than those reported in earlier human pharmacokinetic studies. For example, Østerlie et al. reported a plasma *C*max of 1.3 ± 0.1 µg/mL after a single 100 mg dose of astaxanthin [153]. The markedly higher plasma concentrations reported by Khayyal et al. despite the lower dose highlight the influence of formulation and study conditions on systemic astaxanthin exposure and warrant cautious interpretation when comparing pharmacokinetic results across studies [152,153]. Therefore, the available human pharmacokinetic data do not allow for a conclusion as to whether oral astaxanthin dosing achieves concentrations comparable to those that showed protective effects in vitro.

Astaxanthin bioavailability was also shown to be enhanced by dosing following a meal in the *Haematococcus* algal extract study [154]. These results demonstrate that systemic astaxanthin exposure can be significantly influenced by oral formulation and administration settings. Nevertheless, plasma concentrations cannot be taken to reflect concentrations inside the retina or RPE.

Retina- and RPE-specific concentrations after oral dosing have not been quantified. Ocular accumulation of astaxanthin has been reported in rats after dietary exposure [155]. Without determining the amount of astaxanthin that reaches the retina, oral astaxanthin has demonstrated protective effects in diabetic rats and light-exposed mice [146,156]. Nevertheless, the studies listed here do not yet provide quantitative measures of astaxanthin content in human retinal or RPE tissue. Therefore, whether oral astaxanthin supplementation can result in astaxanthin concentrations in the human RPE that are comparable to those that have protective effects in vitro is still unknown. As a result, measuring astaxanthin directly in retinal and RPE tissues following oral treatment is a crucial translational research goal.

## 9. Therapeutic Implications and Future Perspectives

In an experimental model of macular degeneration, astaxanthin reduced choroidal neovascularization by downregulating inflammatory mediators such as ICAM-1, MCP-1, VEGF, IL-6, VEGFR-1, and VEGFR-2 [144]. However, this CNV study used a neovascular (wet) AMD model and is therefore not directly applicable to dry AMD, which is the primary focus of this review. Astaxanthin’s promise as a treatment for retinal vascular illnesses is supported by the fact that it reduced oxidative stress and inflammation while maintaining retinal structure and function in experimental diabetic retinopathy. Increased production of antioxidant enzymes and decreased NF-κB activity were linked to astaxanthin’s protective properties, indicating that oxidative stress and inflammatory signaling may be modulated [156]. Astaxanthin has been shown in experiments to protect retinal ganglion cells, decrease apoptosis, and maintain visual function in the face of high intraocular pressure and ischemic injury, suggesting wider neuroprotective uses in ocular disorders [157,158]. Astaxanthin deserves more research as a possible adjunctive treatment for retinal degenerative diseases like AMD because of its capacity to lower oxidative stress, suppress inflammatory mediators, prevent pathological neovascularization, and maintain retinal function in a variety of experimental models [144,156,157,158].

A2E loading concentration, blue-light wavelength, intensity, and exposure duration, as well as outcome indicators such as biochemical markers, lipid peroxidation, apoptosis, and retinal function, should all be standardized in subsequent research. The reproducibility and translational relevance of results would be enhanced by such standardization, which would also make quantitative comparison between research easier. As noted above, astaxanthin has not been tested in A2E-specific photooxidation models [95,107]. The following research priorities are particularly important:

1. Validation of A2E-loaded, blue-light-challenged ARPE-19 or primary RPE models with standardized A2E dosing and light-exposure protocols.
2. Head-to-head comparison of astaxanthin with other xanthophylls such as lutein, zeaxanthin, and fucoxanthin within the same A2E photooxidation paradigm to determine relative potency.
3. Pharmacokinetic assessment of astaxanthin delivery across the blood-retinal barrier at concentrations achievable with current oral formulations.
4. Mechanistic determination of whether astaxanthin can directly intercept singlet oxygen generated during A2E photooxidation, thereby competing with A2E for singlet-oxygen addition to the polyene chain of A2E and limiting the formation of oxidized bisretinoids and potentially toxic photodegradation products, versus acting primarily through downstream Nrf2/NF-κB pathway modulation.
5. Placebo-controlled clinical trials of astaxanthin monotherapy rather than combination carotenoid formulations with retinal structural and functional endpoints.
6. Cross-model validation of carotenoid effects in A2E-loaded systems other than ARPE-19 cells, such as D407, RPE-J, primary and polarized RPE cultures, and *Abca4−/−Rdh8−/−* mice, with Nrf2- and NF-κB-dependent readouts as well as mitochondrial dynamics (OPA1, DRP1, OMA1) and ferroptosis markers (Fe2+, lipid peroxidation, SLC7A11-GSH-GPX4).
7. Quantitative measurement of astaxanthin levels in the RPE and neural retina following oral administration in animals and, where human donor tissue is available, in the human eye, with comparison to protective in vitro concentrations to directly quantify the retinal exposure–effect gap identified in Section 8.3.

## 10. Conclusions

The central evidence gap is that astaxanthin has not yet been tested in an A2E-loaded, blue-light-exposed RPE model. Therefore, astaxanthin’s protective effect against A2E-induced photooxidative damage remains an untested hypothesis, even though it has shown antioxidant, anti-inflammatory, and Nrf2/NF-κB-modulating properties in other oxidative-stress and photooxidative models. The available evidence provides a mechanistic rationale for investigating astaxanthin in this context, but it should not be taken as proof of A2E-specific protection or as a therapeutic conclusion.

The available RPE evidence is limited to individual experimental studies, and the strength and consistency of the findings vary across experimental models. Astaxanthin has been reported to activate Nrf2-dependent antioxidant enzymes (HO-1, NQO1, GCLM, and GCLC) in ARPE-19 cells under a H_2_O_2_ challenge [17] and to suppress NF-κB signaling in ARPE-19 cells under high glucose [59]. Each of these effects rests on a single RPE cell study, and no RPE cell study has assessed both pathways together. In vivo data demonstrating decreased oxidative DNA damage and preserved retinal function under prolonged light exposure provide additional evidence of retinal biological activity. However, the animal studies in Table 2 (Supplementary Table S1) have methodological limitations, including unclear or absent reporting of randomization, allocation concealment, blinded outcome assessment, and a priori sample-size estimation, which limits the strength of the in vivo evidence. Importantly, these findings were not obtained in A2E-specific models. Although intriguing, the clinical evidence is still in its early stages. Astaxanthin has not been separated from co-administered lutein and zeaxanthin in previous trials, while retinal exposure remains insufficiently quantified.

Crucially, lutein and zeaxanthin have already been evaluated in A2E-loaded, blue-light-exposed RPE cells, whereas astaxanthin has not yet been tested in this A2E-specific model [95]. Although it is not a direct replacement, the closest surrogate that is now available, all-*trans-*retinal photooxidation, shares with A2E photooxidation the generation of singlet oxygen only; the bisretinoid-specific photooxidation and photodegradation steps present in A2E are absent from the all-*trans*-retinal model. The question of whether astaxanthin works directly on A2E photoproducts or merely on the general oxidative stress pathways that A2E accumulation engages remains unanswered, leaving a unique and consequential evidence gap. Direct testing in an A2E-loaded, blue-light-exposed RPE model is therefore required to determine whether the mechanistic effects translate into A2E-specific protection.

## Figures and Tables

**Table 2:** Experimental models cited in this review for astaxanthin and A2E-mediated photooxidative damage, graded by relevance to A2E.

Model	Injury Method	Dose and Exposure Conditions	Outcome Measures	Nrf2/NF-κB Involvement	A2E Relevance Grade	Ref.
A2E-loaded ARPE-19 cells (human RPE)	Photooxidation of A2E by blue light (430 nm)	A2E (25 µM) administered three times at 48 h intervals before blue-light exposure; cells exposed to 8000 lux for 30 min; astaxanthin not tested in this system	HMOX1 (HO-1) induction; MLL1-dependent chromatin remodeling	NF-κB (RELA) recruitment drives HO-1 induction; Nrf2 not assessed	Direct (validated A2E-loaded, blue-light model)	[57]
ARPE-19 cells + all-*trans*-retinal (at-RAL)	at-RAL loading with blue light-induced phototoxicity	at-RAL (2 µM)-exposed cells; astaxanthin pretreatment (0–2 µM); blue light exposure (450 nm, 7 mW/cm^2^) for 10 min	Singlet oxygen scavenging; reduced oxidative damage; reduced apoptosis	Not assessed	Close surrogate (at-RAL)	[107]
ARPE-19 cells + H_2_O_2_	H_2_O_2_-induced oxidative stress	H_2_O_2_ (200 µM) challenge; astaxanthin (10 µM and 20 µM) pretreatment	ROS levels, cell survival, apoptosis; HO-1, NQO1, GCLC, GCLM expression	Nrf2 activation via PI3K/Akt (phase II enzyme upregulation); NF-κB not assessed	Generic oxidative	[17]
Mouse retina, in vivo	Prolonged light exposure (general photooxidative injury, not A2E-loaded)	Dietary astaxanthin (100 mg/kg) supplementation; White fluorescent light (8000 lux, 3 h)	8-OHdG (oxidative DNA damage); ERG amplitude; outer nuclear layer thickness	Not assessed	Generic oxidative	[146]
ARPE-19 cells + diabetic rat retina	Hyperglycemia; 30 mM glucose in ARPE-19 cells; streptozotocin-induced diabetes in rats	Astaxanthin (1, 5, and 10 µM)	ROS generation, lipid peroxidation, nitric oxide, protein carbonyl, lipid peroxides, SOD, catalase, GPx, GST, NF-κB, TNF-α, ICAM-1, PI3K, p-Akt, and NF-κB nuclear translocation.	NF-κB involved; PI3K/Akt–NF-κB signaling. Nrf2 not assessed/reported	Generic oxidative	[59]
Mitochondrial-mimetic liposomes	Nitrative–oxidative lipid peroxidation (pre-apoptosis model)	Liposome system; astaxanthin (25 µM) treatment	Lipid peroxidation; membrane damage; cytochrome c-mediated pro-apoptotic signaling	Not assessed (cell-free, non-retinal system)	Non-retinal	[76]
A2E-containing ARPE-19 cells	Blue-light-induced photooxidative damage	A2E (10 µM) for 10 days; lutein or zeaxanthin (10 µM) for 3 days after A2E accumulation; blue light (430 ± 20 nm, 2.62 mW/cm^2^) for 10 min; astaxanthin not tested in this system	Photooxidative damage, proteasome activity, ubiquitin conjugates, and expression of inflammation-related genes (MCP-1, IL-8, and CFH)	Inflammation-related gene expression assessed; Nrf2/NF-κB not directly assessed	Direct	[95]
A2E-loaded ARPE-19 cells (mitochondrial dynamics)	Blue-light-emitting diode photooxidation of A2E	A2E (10 and 25 µM); blue LED irradiation (445 ± 18 nm, 4.43 mW/cm^2^); astaxanthin not tested in this system	Viability, ROS, caspase-3 activation, apoptosis; mitochondrial network fragmentation; OPA1, DRP1, and OMA1 expression	Not directly assessed	Direct (A2E-loaded, blue-light model)	[36]
A2E-loaded ARPE-19 cells and C57BL/6 J *Abca4−/−Rdh8−/−* mice	Blue-light photooxidation of A2E (ferroptosis)	A2E (25 and 50 µM) for 48 h, then blue light for 30 min; in vivo blue-light exposure of *Abca4−/−Rdh8−/−* mice; astaxanthin not tested in this system	Fe^2+^ accumulation, ROS, lipid peroxidation, SLC7A11 and GPX4 levels, GSH depletion, ferroptotic cell death; rescue by GSH, deferiprone, or ferrostatin-1	Not directly assessed (GPX4 axis is functionally related to Nrf2-dependent glutathione metabolism)	Direct (A2E-loaded, blue-light model, in vitro and in vivo)	[37]
Human D407 RPE, rat RPE-J, primary bovine RPE, ARPE-19	A2E loading, no blue-light challenge	A2E to aged-RPE-comparable levels; astaxanthin not tested	Lysosomal A2E localization; delayed phagolysosomal degradation; altered cholesterol distribution; lysosomal dysfunction	Not assessed	Direct (A2E) but non-photooxidative; assesses cross-model conservation	[34,35]

**A2E relevance grade:** Direct, A2E-loaded RPE model with blue-light photooxidation; Close surrogate (at-RAL), all-*trans*-retinal photooxidation as a mechanistic proxy for A2E; at-RAL is a retinal precursor rather than a bisretinoid, and the self-sensitized photooxidation step characteristic of A2E is not reproduced in this model; Generic oxidative, non-bisretinoid oxidative or light-induced retinal injury; non-retinal, non-RPE or cell-free system. Direct (A2E) but non-photooxidative, A2E-loaded RPE model studied without a blue-light challenge and included to assess whether A2E effects are conserved across RPE models. Risk of bias for the in vivo entries was appraised with the SYRCLE tool [18] and the CAMARADES checklist [19]. All 10 SYRCLE risk-of-bias domains (Table S1a) and all 10 CAMARADES quality-checklist items (Table S1b) were scored for the in vivo components of the included studies. The reporting of several methodological features was absent or unclear, so the in vivo entries should be interpreted as having unclear to potentially high risk of bias across one or more domains (Supplementary Table S1). The relevance grade reflects biological proximity to A2E pathology only and is not a quality score. **Abbreviations:** 8-OHdG, 8-hydroxy-2′-deoxyguanosine; A2E, *N*-retinylidene-*N*-retinylethanolamine; ARPE-19, human retinal pigment epithelial cell line; at-RAL, all-*trans*-retinal; CFH, complement factor H; ERG, electroretinogram; GCL, glutamate–cysteine ligase; GCLC, glutamate–cysteine ligase catalytic subunit; GCLM, glutamate–cysteine ligase modifier subunit; GPx, glutathione peroxidase; GST, glutathione S-transferase; HO-1, heme oxygenase-1; ICAM-1, intercellular adhesion molecule-1; IL-8, interleukin-8; MCP-1, monocyte chemoattractant protein-1; NF-κB, nuclear factor kappa B; NQO1, NAD(P)H:quinone oxidoreductase 1; Nrf2, nuclear factor erythroid 2-related factor 2; p-Akt, phosphorylated Akt; PI3K/Akt, phosphoinositide 3-kinase/protein kinase B signaling pathway; ROS, reactive oxygen species; SOD, superoxide dismutase; DRP1, dynamin-related protein 1; GPX4, glutathione peroxidase 4; GSH, glutathione; OMA1, overlapping activity with m-AAA protease 1; OPA1, optic atrophy 1; SLC7A11, solute carrier family 7 member 11; D407, human retinal pigment epithelial cell line; LED, light-emitting diode; RELA, RELA proto-oncogene, NF-κB subunit (p65); RPE-J, rat retinal pigment epithelial cell line.

## Data Availability

No new data were created or analyzed in this study. Data sharing does not apply to this article.

## Supplementary Materials

The following supporting information can be downloaded at: https://www.mdpi.com/article/10.3390/antiox15101273/s1. Supplementary Table S1: Judgments follow the SYRCLE risk-of-bias tool for animal studies [18] and the CAMARADES quality checklist [19] and are restricted to the in vivo components of the studies cited in Table 2 [37,59,146]. Table S1a reports scores across all 10 SYRCLE risk-of-bias domains, and Table S1b reports scores across all 10 CAMARADES quality-checklist items. In vitro and cell-free studies were not assessed using these animal-study tools and were evaluated separately according to their A2E relevance, as described in the main text. Table S1a: SYRCLE risk-of-bias domains (D1–D10); Table S1b: CAMARADES quality-checklist items (C1–C10) for the in vivo studies in Table 2.
