# Supplementary material for: Astaxanthin and Related Carotenoids as a Dual Modulator of Nrf2 and NF-κB in Bisretinoid-Induced Retinal Damage: Mechanistic Rationale and Evidence Gaps
Source: Antioxidants (Basel). 2026 Oct 2;15(10):1273. doi: 10.3390/antiox15101273 (PMC13700670; doi:10.3390/antiox15101273)
Supplement: Supplementary file 1 [file antioxidants-15-01273-s001.zip › antioxidants-4576232-supplementary.pdf]

Supplementary Table S1. Domain-level SYRCLE / CAMARADES risk-of-bias judgments for the in vivo studies in Table 2

Judgments follow all 10 SYRCLE risk-of-bias domains for animal studies [18] (Table S1a) and all 10 CAMARADES quality-checklist items [19] scored as Yes, No, or Unclear (Table S1b), restricted to the in vivo components of the studies cited in Table 2, consistent with the Table 2 footnote and Section 2.3. In vitro and cell-free studies in Table 2 are not appraised with these tools and are graded only by A2E relevance, as stated in the main text.

'Unclear' is used alone in the cells throughout this table and indicates that the item could not be determined from the published methods as available to the reviewers; it does not indicate the study did not perform the procedure. 'Low' (Table S1a) and 'Yes' (Table S1b) indicate the reported information supports that judgment; 'No' (Table S1b) indicates the reported information does not support that judgment.

For Yang et al. [37], the full open-access text (including the Declarations section) was read in full; its Unclear and Yes/No judgments above reflect that complete read. For Otsuka et al. [146] and Janani et al. [59], the full texts remained inaccessible beyond the published abstract/methods (J-STAGE access restriction and journal paywall, respectively), so Unclear judgments for those two rows reflect the information available in the accessible text rather than a confirmed absence.

Table S1a. SYRCLE risk-of-bias domains (D1–D10)

Study (ref.)	D1	D2	D3	D4	D5	D6	D7	D8	D9	D10
Otsuka et al. 2013 [146] dietary astaxanthin, light-induced retinal damage, mouse	Unclear	Low, male albino ddY mice, 8–10 weeks old, were described	Unclear	Unclear	Unclear	Unclear	Unclear	Unclear	Unclear	Unclear
Janani et al. 2022 [59] streptozotocin-induced diabetic rat retina (in vivo component)	Unclear	Low, male Wistar rats, 8 weeks old and 90–100 g, were described; diabetes was confirmed by FBG >200 mg/dL	Unclear	Unclear	Unclear	Unclear	Unclear	Low; 24 rats were allocated to three groups of eight, and no exclusions/dropouts were reported in the animal Methods	Unclear	Unclear
Yang et al. 2025 [37] C57BL/6J *Abca4*$^{-/-}$*Rdh8*$^{-/-}$ mice, blue-light exposure (in vivo component)	Unclear	Low, 3-month-old mice and their strains/genotypes were specified	Unclear	Unclear	Unclear	Unclear	Unclear	Unclear	Unclear	Unclear

D1 Sequence generation / random allocation • D2 Baseline characteristics similar • D3 Allocation concealment • D4 Random housing • D5 Blinding of caregivers/investigators • D6 Random outcome assessment • D7 Blinded outcome assessment • D8 Incomplete outcome data addressed • D9 Selective outcome reporting • D10 Other sources of bias

Table S1b. CAMARADES quality-checklist items (C1–C10)

Study (ref.)	C1	C2	C3	C4	C5	C6	C7	C8	C9	C10
Otsuka et al. 2013 [146]	Yes (peer-reviewed, J Pharmacol Sci)	Yes (25 ± 1.5 °C maintained during light exposure)	Unclear	Unclear	Unclear	Yes (anesthetic used only for terminal ERG, after	No	Unclear	Yes (Gifu Pharm. Univ. IACUC; ARVO Statement)	Unclear

Study (ref.)	C1	C2	C3	C4	C5	C6	C7	C8	C9	C10
						unanesthetized light exposure)				
Janani et al. 2022 [59]	Yes (peer-reviewed, Eur J Pharmacol)	Unclear	Unclear	Unclear	Unclear	Unclear	Yes (STZ-diabetic rat is a disease-relevant comorbidity model)	Unclear	Yes (IAEC approval No. 98/2017)	Unclear
Yang et al. 2025 [37]	Yes (peer-reviewed, open access, Cell Mol Biol Lett)	Unclear	Unclear	Unclear	Unclear	Unclear	Yes (*Abca4−/−Rdh8−/−* is an A2E-relevant genetic model)	Unclear	Yes (IACUC, Xiamen University Laboratory Animal Center)	Yes (Declarations state competing interests)

C1 Peer-reviewed publication • C2 Statement of temperature control • C3 Random allocation to group • C4 Allocation concealment • C5 Blinded assessment of outcome • C6 Anesthetic without marked intrinsic neuroprotective/toxic activity • C7 Appropriate animal model • C8 Sample-size calculation • C9 Compliance with animal welfare regulations (ethical approval) • C10 Statement of conflict of interest

Notes

Study (ref.)	Notes
Otsuka et al. 2013 [146]	In vivo animal components only. This was a non-A2E, light-induced retinal injury model and was classified as Generic oxidative for A2E relevance in Table 2. The authors reported animal-care approval and ARVO guidance.
Janani et al. 2022 [59]	In vivo diabetic-rat arm only; the ARPE-19 hyperglycemia arm of this study is in vitro and is graded only by A2E relevance (non-bisretinoid, generic oxidative model), not by SYRCLE/CAMARADES.
Yang et al. 2025 [37]	In vivo arm only; the A2E-loaded ARPE-19 arm of this study is in vitro and is graded by A2E relevance/mechanistic directness, not by SYRCLE/CAMARADES.

Abbreviations: ERG, electroretinogram; OCT, optical coherence tomography; 8-OHdG, 8-hydroxy-2′-deoxyguanosine; FBG, fasting blood glucose; IAEC, Institutional Animal Ethics Committee; IACUC, Institutional Animal Care and Use Committee; ARVO, Association for Research in Vision and Ophthalmology; STZ, streptozotocin.
